# Research on the Development of Digital Creative Sports Industry Based on Deep Learning

**DOI:** 10.1155/2022/7760263

**Published:** 2022-01-30

**Authors:** Junmeng Chen, Shaofeng Xu

**Affiliations:** Sangmyung University, Seoul 03016, Republic of Korea

## Abstract

The core of the digital entrepreneurial sports culture creative industry lies in innovation, which emphasizes the new impetus brought by the digital entrepreneurial sports culture to the social economy. The digital entrepreneurial sports cultural creative industry is rooted in the cultural creative industry. The digital entrepreneurial sports cultural creative industry is also an important part of the sports industry, and its development highly depends on the development of the sports industry. The digital entrepreneurial sports cultural creative industry has the characteristics of both the sports industry and the cultural creative industry. This paper uses the deep learning technology to study the development of the digital creative sports industry and build an intelligent model. Moreover, this paper assigns weights to the input multidimensional features, extracts the most relevant data features, and analyzes the performance of the proposed model through simulation experiments. From the experimental analysis results, we can see that the model proposed in this paper has certain practicality.

## 1. Introduction

The sports culture creative industry has high cultural connotation in the sports industry, and it respects people's creativity and thinking ability. Moreover, it is the sum of the activities that have been industrialized through the support of science, technology, and market-oriented operation with the main characteristics of highlighting creativity and innovation capabilities. The sports culture creative industry focuses on innovation, and it emphasizes the new economic concepts and huge driving forces brought by sports culture to the society [1]. The development of the sports culture creative industry is highly dependent on the sports culture market and the sports culture mechanism, and the development of the sports culture creative industry is more dependent on the development of the sports industry. At the same time, it can promote the development of the sports industry, and it will become a new economic growth point for the development of the sports industry. With the establishment and development of China's socialist market economic system, the sports culture industry as a low-carbon and environmentally friendly “sunrise industry” in the national economy has gradually become a new economic growth point [2].

The creative industry is a new type of transnational, cross industry, cross department and cross field reorganization or innovation industry under the background of globalization, based on the spiritual, cultural and entertainment needs of the consumption era, supported by high-tech means, dominated by new communication modes such as network, and characterized by the comprehensive combination of culture, art and economy. It is supported by high-tech means, led by the latest communication methods such as the Internet, and characterized by the comprehensive combination of culture, art, and economic technology [3]. It is a composite concept that takes creative innovation as the core and the operation of knowledge capital as a means to control the entire process of industrial development such as production, communication, circulation, and consumption. Moreover, it is an emerging industry cluster that provides cultural, artistic, spiritual, psychological, and entertainment products to the public. Other terminology used to describe creative industry includes creative economy It mainly refers to those enterprises that rely on individual creativity, skills, and talents to seek development ideas and development motivation, as well as emerging industries that create potential wealth and employment opportunities through the development of intellectual property rights and the transformation of old industries [4].

This article uses deep learning technology to study the development of the digital creative sports industry, builds an intelligent model, and combines simulation data for model research to provide a theoretical reference for the subsequent development of the digital creative sports industry.

## 2. Related Work

There are abundant theoretical studies on creative industries in foreign academic circles. Literature [5] pointed out that the fundamental driving force of modern economic development is innovation, and the key to innovation is the production, dissemination, and use of knowledge and information. Literature [6] pointed out that new ideas will derive endless new products, new markets, and new opportunities for wealth creation. New ideas are the driving force behind the economic growth of a country. Literature [7] believes that the creative industry uses the creativity of the human brain to create wealth and employment opportunities, and defines the creative industry as an economic sector whose products are protected by the intellectual property law. The definition in [8] extends the connotation of the creative industry and incorporates patent research and development activities belonging to various departments in the natural sciences into the creative industry, effectively solving the problem of the separation of science and culture and art in creative activities. Literature [9] pointed out that the importance of culture lies in being a platform and resource for creativity. Literature [10] believes that there is a fairly new category of academic, policy, and industrial discourse in the creative industry. It can capture the dynamics of a large number of new-economy companies, which cannot be achieved by words such as “art,” “media,” and “cultural industry.” Literature [11] puts forward the driving factors and limiting factors in the development of creative economy. It also pointed out that the sudden emergence of creativity in the contemporary economy indicates the rise of a professional class. Literature [12] puts forward the characteristics of contemporary cultural and creative industries and “artistic commercial approach.”

Literature [13] put forward the necessity of developing creative industries and, for the first time, defined the cultural creative industry from the perspective of the industrial chain: realization of property rights or consumption is a transaction feature that provides cultural experience for the public. Literature [14] analyzes the content of creative economy, the creative value, and the development of sports culture and creative industries and discusses the development of sports culture and creative industries. and discusses the necessity of the development of sports culture and creative industry. Literature [15] pointed out that in order to achieve faster and better development of the sports industry, it is necessary to further tap the industrial development potential and innovate the industrial development model, especially to integrate cultural and creative elements into the development of the sports industry and to adopt sports cultural creativity. The industry is the leading one, and it drives the development of other related industries. Literature [16] analyzes the development and improvement of the sports culture creative industry and draws the feasibility of the development of the sports culture creative industry. Paper [17] pointed out that the development and improvement of the sports cultural creative industry will directly affect the sustainable development of the sports industry. By analyzing the feasibility of the development of the sports cultural creative industry, it summarizes the main problems in the current development of the sports cultural creative industry and puts forward recommendations accordingly. Literature [18] pointed out the development status of the sports cultural creative industry and provided corresponding suggestions for rationally planning the development pattern of the sports cultural creative industry; formulating a scientific, effective, and feasible development action plan; and promoting the sustainable, healthy, and stable development of the sports cultural creative industry.

## 3. Data Processing of Digital Creative Sports Industry Based on Deep Learning

**Figure 1:**
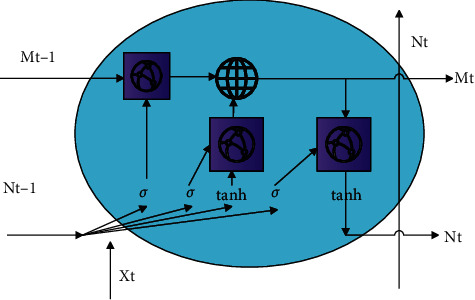
LSTM network structure diagram.

**Figure 2:**
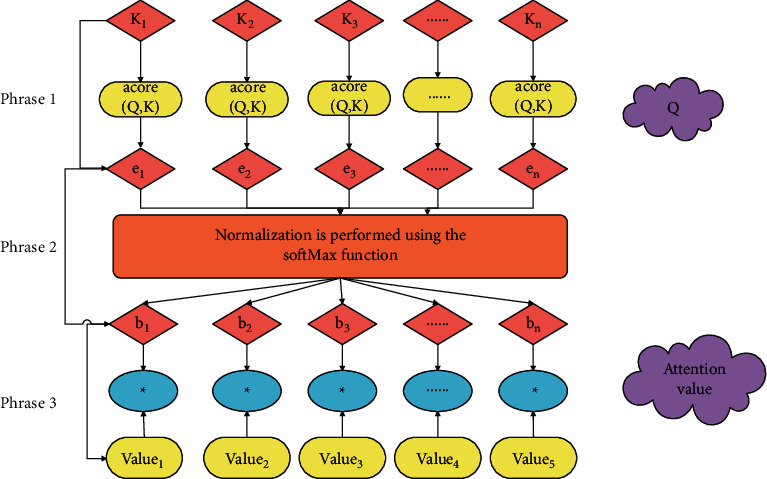
Diagram of the calculation process of the attention mechanism.

**Figure 3:**
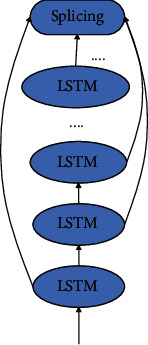
Enhanced LSTM structure diagram.

**Figure 4:**
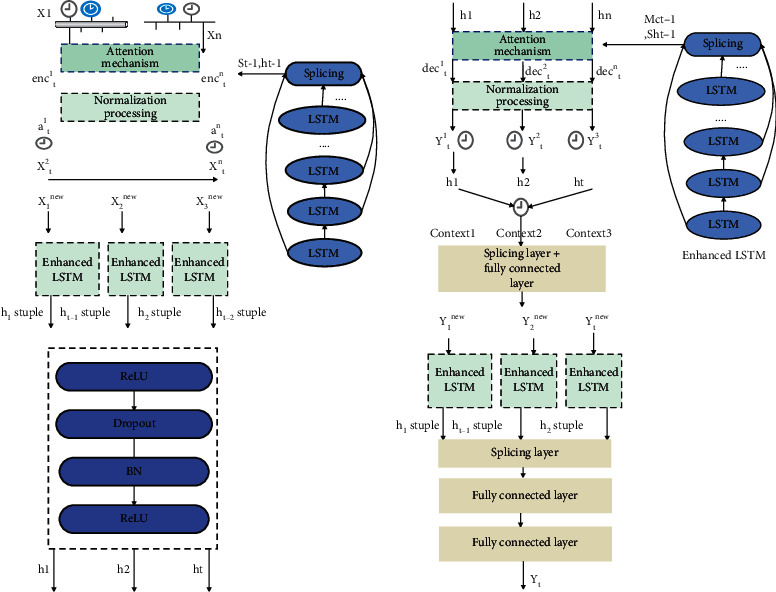
Attention prediction model based on enhanced LSTM. (a) Encoding stage. (b) Decoding stage.

**Figure 5:**
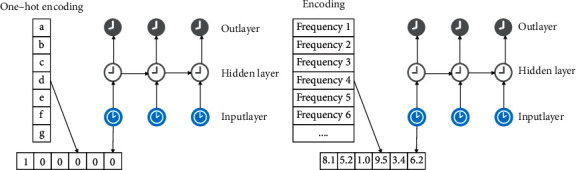
Schematic diagram of coding. (a) Data processing 1. (b) Data processing 2.

**Figure 6:**
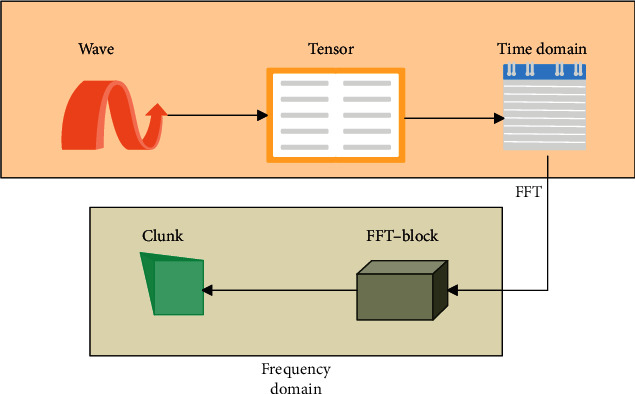
Schematic diagram of data preprocessing.

Since the RNN network has the problem of gradient disappearance and dispersion in solving the long-term dependence problem, the LSTM neural network is proposed to solve the problem of gradient disappearance and gradient dispersion. The LSTM network adds a gating mechanism on the basis of the RNN network to combine short-term memory with long-term memory, which solves the problem of gradient disappearance to a certain extent. The gating mechanism is mainly composed of three gates, which are input gates, forget gates, and output gates. At the same time, the LSTM network also has a memory unit. The input gate is mainly used to control the storage of the current digital creative sports industry data to the memory unit. The forget gate is used to control the digital creative sports industry data of the memory unit at the previous moment and save it to the memory unit at the current moment. Moreover, the output gate mainly controls whether the digital creative sports industry data in the current memory unit is useful to the hidden unit. If it is useful, it passes to the hidden unit. The function of the memory unit is to comprehensively consider the current input digital creative sports industry data and the digital creative sports industry data of the memory unit at the previous moment. Next, this article combines Figure 1 and (1)–(6) to introduce the working principle of the LSTM network in detail. The following are the calculation formulas that represent the various gates and state mechanisms of the LSTM neural network [19].

(1) $$\begin{array}{c} i_{t}^{\left(l\right)}=\sigma \left(W_{ix}^{\left(l\right)}x_{t}^{\left(l-1\right)}+W_{iN}^{\left(l\right)}N_{t-1}^{\left(l\right)}+b_{i}^{\left(l\right)}\right), \end{array}$$

(2) $$\begin{array}{c} g_{t}^{\left(l\right)}=\tanh\left(W_{gx}^{\left(l\right)}x_{t}^{\left(l-1\right)}+W_{gN}^{\left(l\right)}N_{t-1}^{\left(l\right)}+b_{g}^{\left(l\right)}\right), \end{array}$$

(3) $$\begin{array}{c} f_{t}^{\left(l\right)}=\sigma \left(W_{fx}^{\left(l\right)}x_{t}^{\left(l-1\right)}+W_{fN}^{\left(l\right)}N_{t-1}^{\left(l\right)}+b_{f}^{\left(l\right)}\right), \end{array}$$

(4) $$\begin{array}{c} o_{t}^{\left(l\right)}=\sigma \left(W_{ox}^{\left(l\right)}x_{t}^{\left(l-1\right)}+W_{oN}^{\left(l\right)}N_{t-1}^{\left(l\right)}+b_{o}^{\left(l\right)}\right), \end{array}$$

(5) $$\begin{array}{c} M_{t}^{\left(l\right)}=g_{t}^{\left(l\right)}⊙i_{t}^{\left(l\right)}+M_{t-1}^{\left(l\right)}⊙f_{t}^{\left(l\right)}, \end{array}$$

(6) $$\begin{array}{c} N_{t}^{\left(l\right)}=\tanh\left(M_{t}^{\left(l\right)}\right)⊙o_{t}^{\left(l\right)}. \end{array}$$

In these formulas, *i*_*t*_^(*l*)^ represents the input state of the *t*-th time step of the *l*-th layer, *f*_*t*_^(*l*)^ represents the reserved state of the *t*-th time step of the *l*-th layer, *o*_*t*_^(*l*)^ represents the output state of the *t*-th time step of the *l*-th layer, *g*_*t*_^(*l*)^ represents the input of the LSTM unit of the *t*-th time step of the *l*-th layer, *g*_*t*_^(*l*)^ represents the state of the storage unit at the *t*-th time step of the *l*-th layer, and *g*_*t*_^(*l*)^ represents the output of the hidden unit at the *t*-th time step of the *l*-th layer. *σ* represents the sigmoid activation function, whose expression is shown in (7), and its output value is within [0, 1]. tanh is the activation function, whose expression is shown in (8), and its output value range is [−1, 1]. W represents the weight matrix, and b represents the bias; they are used for the connection between the input layer, hidden layer, and output layer. *i*, *g*, *o* represent input gate, forget gate, and output gate, respectively. ⊙ stands for Hadamard multiplication. Through the sigmoid function, the output values of the three gates are all within [0, 1] [20].

(7) $$\begin{array}{c} \mathrm{sigmoid}\left(x\right)=\frac{1}{1+e^{-x}}, \end{array}$$

(8) $$\begin{array}{c} \tanh\left(x\right)=\frac{e^{x}-e^{-x}}{e^{x}+e^{-x}}. \end{array}$$

The attention mechanism in neural networks is a method proposed by Bahdanau et al. in 2014 to solve machine translation problems, due to the inadequacy of the traditional model in machine translation; that is, when the model predicts the current output word, it only considers the current hidden state and the last output word. This requires the model to compress all sequences into a fixed-length vector when receiving the input sequence. The length of the vector will therefore make the model appear powerless when facing long sentences. As the input sequence grows, the digital creative sports industry data of the words in the front of the sentence will be seriously lost. At the same time, during decoding, the digital creative sports industry data corresponding to the current predicted words are basically lost, which seriously affects the prediction performance of the model. In order to solve the above-mentioned problems, an attention mechanism was created. The essence of the attention mechanism is to retain the contextual digital creative sports industry data and location digital creative sports industry data of the input word corresponding to the current prediction word. After the input sequence is encoded into a fixed vector, the decoding stage will automatically extract the current data from the fixed vector to predict the useful digital creative sports industry data for words. At the same time, the attention mechanism can effectively solve the computational bottleneck problem of convolutional neural network and the problem of neural network inexplicability. It is a mechanism for improving the effect of encoder and decoder models based on RNN (LSTM or GRU). Since the development of the attention mechanism, many types of attention models have been derived, such as soft attention models, hard attention models, hierarchical attention models, and self-attention models. In the next section, we mainly introduce the basic principles of the attention mechanism.

The power of the attention mechanism is that it gives the model the ability to differentiate data differentially and assigns and assigns different weights to different parts of the input digital creative sports industry data. It solves the problem of compressing the input digital creative sports industry data to a fixed-length vector in the Seq2Seq model, ignoring the actual length of the input digital creative sports industry data, and assigning the same weight to different parts of the input digital creative sports industry data. The attention mechanism is a mechanism to improve the performance of RNN-based encoder and decoder models. The specific calculation process of the attention mechanism can be abstracted and summarized into three processes. Next, according to Figure 2, the three processes are introduced in detail:

(1) We assume that the main part is Source and imagine its constituent elements as a series of key-value pairs such as <*K*, value>, that is, the data structure of the digital creative sports industry. When we give a certain element *Q* in Target, we first calculate the similarity or correlation between *О* and each *K* and mark it as *e*_*i*_(∀*i*=1,2,3, ..., *n*). This process is stage one shown in Figure 2, and it is as shown in the following formula [21]:
  (9) $$\begin{array}{c} e_{i}=\mathrm{score}\left(Q,K_{i}\right)\left(\forall i=1,2,3,\ldots ,n\right). \end{array}$$
Among them, score is the alignment function, and the most common calculation method is to perform the vector product of the two; that is, score(*Q*, *K*_*i*_)=*Q* · *K*_*i*_.
(2) After obtaining the similarity between *O* and each *K*, it is necessary to normalize these similarity values. In this way, the calculated original score can be sorted into a probability distribution where the sum of the weights of all elements is 1. At the same time, by assigning corresponding weights to each *K*, the important elements are more prominent. This process is stage two and is shown in the following formula:
  (10) $$\begin{array}{c} b_{i}=\frac{\exp\left(e_{i}\right)}{\sum_{i=1}^{n}\exp\left(e_{i}\right)}\;\left(\forall i=1,2,3,\ldots ,n\right). \end{array}$$
(3) After the weight of each *K* element is obtained, the value corresponding to each *K* needs to be multiplied and summed with the corresponding weight, which is stage three. The value obtained by the weighted summation is the attention value corresponding to *Q*, as shown in the following formula:

(11) $$\begin{array}{c} \text{attention value}=\sum_{i=1}^{n}b*\text{value}. \end{array}$$

The above three stages are the essential ideas of the attention mechanism.

Due to its intuitiveness, versatility, and interpretability, the attention model has become an active area of research by scholars at home and abroad, and the research results have been effectively verified at the enterprise level. Attention mechanism models are widely used in different types of deep learning tasks such as machine translation, question answering, sentiment analysis, part-of-speech tagging, dialogue systems, text classification, summary generation, recommendation systems, and image recognition. It is currently one of the worthiest of in-depth research and in-depth understanding of the technology.

The enhanced LSTM structure is shown in Figure 3. It is composed of multiple basic LSTMs, but it is different from the stacked LSTM structure. When we design the enhanced LSTM structure, we bind the hidden unit state and memory state of each layer of LSTM unit at a certain moment together. In this way, not only can the hidden unit state data and memory state data of the current layer be fully utilized but also the hidden unit state data and memory state data of all layers other than the current layer can be used as auxiliary input digital creative sports industry data to improve the model network's ability to understand the time series. At the same time, the hidden unit status digital creative sports industry data and memory status digital creative sports industry data of all layers except the current layer are used as auxiliary input digital creative sports industry data. This also improves the model network's ability to predict time series digital creative sports industry data.

The attention prediction model based on enhanced LSTM proposed in this paper is mainly composed of two parts: the encoding stage and the decoding stage. Each stage uses the enhanced LSTM proposed in this article as the basic encoder and decoder.  Figure 4(a) shows the coding stage of the prediction model. The encoding stage is mainly employed to use the attention mechanism to calculate the weights of the input multidimensional time series digital creative sports industry data features, so as to extract the most relevant features.  Figure 4(b) shows the decoding stage of the prediction model. The decoding stage mainly distributes attention weights to different units of the hidden layer generated in the encoding stage to extract the most relevant hidden units. In order to simplify the description, this paper defines (1) to (6) as the function enhanced LSTM. Its function is to output the current hidden state and the tuple of the current hidden state and memory state. Next, we will introduce the main details of each stage.

### 3.1. Coding Stage

First, we give a multidimensional time series data set pair (*X*, *y*):

(12) $$\begin{array}{c} X=\left(x_{1},x_{2},x_{3},\ldots ,x_{t},\ldots ,x_{T}\right),\\ y=\left(y_{1},y_{2},y_{3},\ldots ,y_{t},\ldots ,y_{T-1}\right). \end{array}$$

Among them, *x*_*t*_ ∈ *R*^*n*^ is the number of input multidimensional time series data features, and *T* is the size of the sliding window, *y*_*t*_ ∈ *R*; the state of the hidden unit at time *t*−1 is as shown in (1).

(13) $$\begin{array}{c} h_{t-1}=\left[{\text{stuple}}_{t-1}^{1}\left[h\right];{\text{stuple}}_{t-1}^{2}\left[h\right];\ldots ;{\text{stuple}}_{t-1}^{p}\left[h\right]\right]. \end{array}$$

Among them, *p* is the number of layers in the enhanced LSTM. In the two stages of the prediction model in this paper, the value of *p* is set to 3. stuple, [*h*], le[1, *p*] represent the output of the hidden state of the *l*-th layer at time *r*−1. At the same time, the algorithm binds the hidden unit states of all layers together to get the final hidden unit state at time *r*−1. In the same way, this paper can splice the memory cell states of all layers together to get the final memory cell state at time *t*−1, as shown in the following formula:

(14) $$\begin{array}{c} s_{t-1}=\left[{\text{stuple}}_{t-1}^{1}\left[s\right];{\text{stuple}}_{t-1}^{2}\left[s\right];\ldots ;{\text{stuple}}_{t-1}^{p}\left[s\right]\right]. \end{array}$$

Among them, stuple_*t*−1_^1^[*s*], *l* ∈ [1, *p*], means the state of the memory cell of the first layer at time *t*−1. In order to facilitate the use of the enhanced LSTM function, this article needs to save the hidden unit state and memory unit state obtained at *t*−1 above in the form of a tuple as shown in (15) and pass it to the function enhanced LSTM:

(15) $$\begin{array}{c} \text{stuple}=\text{tuple}\left(h_{t-1},s_{t-1}\right). \end{array}$$

In this paper, LSTM is transformed, so that it not only has the ability of LSTM network to deal with the disappearance of gradients, but also has better ability to handle high-dimensional data than LSTM.

We assume that the *q*-th feature data in the input multidimensional time series data is as follows:

(16) $$\begin{array}{c} x^{q}={\left(x_{1}^{q},x_{2}^{q},\cdots ,x_{T}^{q}\right)}^{T}. \end{array}$$

Next, this paper uses the enhanced LSTM-based attention mechanism to encode it at time *t* as follows:

(17) $$\begin{array}{c} \mathrm{en}c_{t}^{q}=v_{\mathrm{enc}}^{T}\tanh\left(W_{\mathrm{enc}}\left[h_{t-1};s_{t-1}\right]+Z_{\mathrm{enc}}x^{q}\right). \end{array}$$

Among them, *v*_enc_^*T*^ ∈ *R*^*T*^, *W*_enc_ ∈ *R*^*T*×2*m∗p*^, *Z*_enc_ ∈ *R*^*T*×*T*^ are the number of hidden layer units, which are all parameter matrices obtained through model training, and enc_*t*_^*q*^ represents the similarity between the hidden state vector at time *I* and the *q*-th feature of the input multidimensional time series data. Furthermore, this paper calculates the attention weight of the *q*-th feature in all features at time *t* based on the similarity, as shown in the following formula:

(18) $$\begin{array}{c} \alpha_{1}^{q}=\mathrm{soft}\max\left(\mathrm{en}c_{t}^{q}\right)=\frac{\exp\left(\mathrm{en}c_{t}^{q}\right)}{\sum_{q=1}^{n}\exp\left(\mathrm{en}c_{t}^{q}\right)}. \end{array}$$

In this paper, we can obtain the attention weight of the remaining features at time *t* according to the above method, as described in the following formula:

(19) $$\begin{array}{c} \alpha_{t}=\left(\alpha_{t}^{1},\alpha_{t}^{2},\ldots ,\alpha_{t}^{n}\right). \end{array}$$

When the weight of each feature of the input multidimensional time series data is obtained in this paper, the original data and the corresponding weight are multiplied to obtain the new input data at time *t*, as shown in the following formula:

(20) $$\begin{array}{c} x_{t}^{\mathrm{new}}={\left(\alpha_{t}^{1}x_{t}^{1},\alpha_{t}^{2}x_{t}^{2},\ldots ,\alpha_{t}^{n}x_{t}^{n}\right)}^{T}. \end{array}$$

Next, we can get the hidden unit state *h*_*t*_ at time *t* according to the following formula and use it as the input of the decoding stage:

(21) $$\begin{array}{c} h_{t},\text{stuple}=\text{EnhancedLSTM}\left(x_{t}^{\mathrm{new}},\text{stuple}\right). \end{array}$$

Among them, *h*_*t*_ ∈ *R*^*m∗p*^. In order to improve the prediction performance of the model and prevent the occurrence of model overfitting, this paper uses Rectified Linear Unit (ReLU), Batch Normalization (BN) and Dropout technology to solve the overfitting problem within a time window.

In the model in this article, ReLU is selected as the activation function of the prediction model. For deep networks, when functions such as sigmoid and tanh are used as activation functions, the derivation is more complicated and the amount of calculation is large. When ReLU is used, the entire calculation process becomes simple, and the convergence speed of the network model is accelerated. Its expression is as follows:

(22) $$\begin{array}{c} \mathrm{ReLU}\left(x\right)=\left\{\begin{array}{cc} x & \mathrm{if}\;x>0,\\ 0 & \text{otherwise}. \end{array}\right) \end{array}$$

Therefore, the calculation will be very convenient when deriving the derivative, as shown in the following formula:

(23) $$\begin{array}{c} {\mathrm{ReLU}}^{\prime }\left(x\right)=\left\{\begin{array}{cc} 1 & \mathrm{if}\;x>0,\\ 0 & \text{otherwise}. \end{array}\right) \end{array}$$

At the same time, when using sigmoid and tanh functions for backpropagation, for deep networks, the problem of gradient disappearance is prone to occur, and the learning and training of deep networks cannot be completed. It can be seen from (22) that ReLU will make the output of some neurons 0, which makes the network sparse, reduces the interdependence between parameters, and can effectively avoid the occurrence of overfitting.

This article still uses enhanced LSTM in the decoding stage to decode the input digital creative sports industry data generated in the encoding stage. Similar to the encoding stage, when decoding the digital creative sports industry data at time *r*, it is necessary to know the hidden unit state *hs* and memory state *mc* at time *t*−1. Like the idea of the coding stage, the state of the hidden units of different layers at the same time needs to be spliced, as shown in the following formula:

(24) $$\begin{array}{c} hs_{t-1}=\left[{\text{stuple}}_{t-1}^{1}\left[\mathrm{hs}\right];{\text{stuple}}_{t-1}^{2}\left[\mathrm{hs}\right];\cdots ;{\text{stuple}}_{t-1}^{p}\left[\mathrm{hs}\right]\right]. \end{array}$$

Among them, stuple_*t*−1_^*i*^[hs], *i* ∈ [1, *p*], represents the hidden unit state of the *i*-th layer at time *t*−1, hs_*t*−1_ ∈ *R*^*m∗p*^. Similarly, at time *t*−1, the state of the memory unit is as shown in the following formula:

(25) $$\begin{array}{c} mc_{t-1}=\left[{\text{stuple}}_{t-1}^{1}\left[\mathrm{mc}\right];{\text{stuple}}_{t-1}^{2}\left[\mathrm{mc}\right];\cdots ;{\text{stuple}}_{t-1}^{p}\left[\mathrm{mc}\right]\right]. \end{array}$$

Among them, stuple_*t*−1_^*i*^[mc], *i* ∈ [1, *p*], represents the state of the *i*-th memory cell at time *t*−1, mc_*t*−1_ ∈ *R*^*m∗p*^.

Next, the algorithm needs to decode the digital creative sports industry data at time *t*. This article first needs to calculate the similarity between the digital creative sports industry data output by the encoder in a time window and the state of the hidden unit at time *t*, which is shown in the following formula:

(26) $$\begin{array}{c} \mathrm{de}c_{t}^{j}=v_{\mathrm{dec}}^{T}\tanh\left(w_{\mathrm{dec}}\left[hs_{t-1};mc_{t-1}\right]+Z_{\mathrm{dec}}h_{j}\right). \end{array}$$

Among them, *v*_dec_ ∈ *R*^*m*^, *w*_dec_ ∈ *R*^*m*×2*m∗p*^, *Z*_dec_ ∈ *R*^*m*×*m∗p*^. They are all parameter matrices obtained through model learning and training. dec represents the similarity or correlation between the encoding phase at time *j* and the hidden unit state at time *t*, and *j* ∈ [1, *T*].*T* is the size of a time window.

This article needs to calculate the corresponding attention weight based on the above-mentioned similarity, as shown in the following formula:

(27) $$\begin{array}{c} y_{t}^{j}=\mathrm{soft}\max\left(\mathrm{de}c_{t}^{j}\right)=\frac{\exp\left(\mathrm{de}c_{i}^{j}\right)}{\sum_{j=1}^{T}\exp\left(\mathrm{de}c_{i}^{j}\right)}. \end{array}$$

In the same way, the algorithm can obtain the weights of hidden units at different moments in a time window and further perform a weighted summation of all hidden units in a time window at time *t* to obtain context, as shown in the following formula:

(28) $$\begin{array}{c} {\text{context}}_{t}=\sum_{i=1}^{T}y_{t}^{i}h_{i}. \end{array}$$

This article first calculates context_*t*_ at time *t* in a time window *T* each time. Among them, *t* ∈ [1, *T*]. Then, it is spliced with the corresponding real target value to predict the value of time *T*, as shown in the following formula:

(29) $$\begin{array}{c} y_{t-1}^{\mathrm{new}}=\text{Dense}\left(\left[y_{t-1};{\text{context}}_{t-1}\right]\right). \end{array}$$

Among them, *y*_*t*−1_ is the true value at time *t*−1. Here, a fully connected layer (Dense) is used to flatten the spliced data to meet the input format. Finally, this paper can use the following formulas to find the hidden unit state hs_t−1_ at time *t*.

(30) $$\begin{array}{c} \text{stuple}=\text{tuple}\left(hs_{t-1},mc_{t-1}\right), \end{array}$$

(31) $$\begin{array}{c} {\text{hs}}_{t-1},\text{stuple}=\mathrm{Enhanced}\;\mathrm{LSTM}\left(y_{t-1}^{\mathrm{new}},\text{stuple}\right). \end{array}$$

Through the above steps, the hidden unit state hs_*T*_ and the memory unit state mc_*T*_ at time *T* in the decoding stage can be obtained Finally, this paper uses two fully connected layers to get the final predicted value ${\hat{y}}_{T}$ at time *T*, as shown in the following formula:

(32) $$\begin{array}{c} {\hat{y}}_{T}=\mathrm{Dense}\left(\mathrm{Dense}\left(\left[hs_{T};\mathrm{contex}t_{T}\right]\right)\right). \end{array}$$

The above is the detailed process of the enhanced LSTM-based attention prediction model proposed in this paper in the encoding stage and the decoding stage. In the next section, a two-stage pseudocode implementation is given.

## 4. Research on the Development of Digital Creative Sports Industry Based on Deep Learning

The model in this article is mainly used for research on the development of digital creative sports industry. We adopt a preprocessing method to make the preprocessed data file suitable for Char-RNN model. The preprocessing method is based on the transformation of the time domain and the frequency domain of the signal and realizes the transformation from continuous data to discrete data. Then, each bit of the input vector in the model represents a specific frequency, and the number at that position represents the amplitude corresponding to this frequency, that is, the contribution value of the current frequency. The coding diagram is shown in Figure 5.

The data preprocessing process is shown in Figure 6.

**Figure 7:**
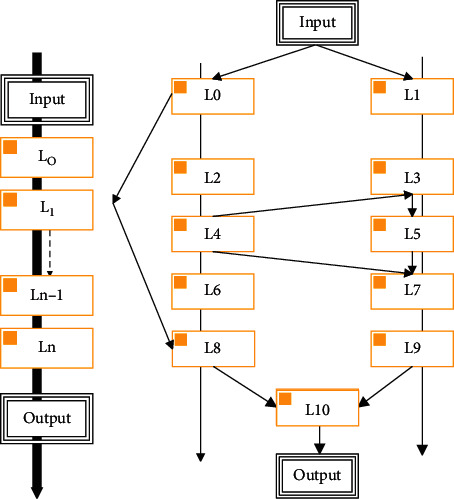
The complex structure of multibranch and jump connections. (a) Chain structure. (b) Complex structure with multiple branches and jump connections.

Neural Architecture Search (NAS) is an automated process of neural network architecture engineering. It was originally proposed in the hope that the learning and design of neural network architecture are the same as deep learning for feature extraction, so that the learned network model has better performance than the artificially designed model, and it does not need to consume a lot of expert experience. Therefore, NAS is the next problem to be solved in the field of machine learning automation. NAS can be regarded as a subfield of AutoML and has many intersections with hyperparameter optimization and metalearning.  Figure 7 shows examples of different architecture spaces.

**Figure 8:**
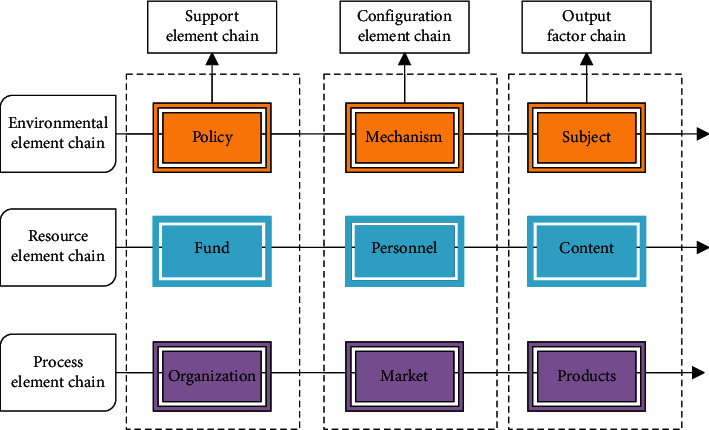
Ecological model of digital creative sports industry.

On the basis of the analysis of the elements and characteristics of the digital creative sports industry, the scientific popularization industry circular ecological model is constructed from the perspectives of systems and ecology. The digital creative sports industry ecological model is an idealized abstract or simplified representation of the constituent elements and mutual relationships of the popular science industry ecosystem. The digital creative sports industry is a unified whole of a series of related resources and resource allocation mechanisms. Therefore, the digital creative sports industry needs to obtain resources from the social system, especially the core resources that have a driving effect, to develop. Under the effect of the market mechanism, these basic resources are appropriately supplied to provide a necessary guarantee system for the harmonious and sustainable development of the popular science industry. From the perspective of industrial ecology, this article sorts out the key factors of the digital creative sports industry, and based on the orientation, scarcity, and circulation of the elements, it is divided into three horizontal element chains and three element rings vertically. Based on this, an ecological model of the popular science industry is established, and the dynamic mechanism of the development of the popular science industry is discussed, as shown in Figure 8.

After constructing a digital creative sports industry development model based on deep learning, the performance of the model is studied. This paper explores the practical effects of the model in terms of industrial market analysis, industrial data processing, and industrial development decision-making and combines the data obtained from the network for simulation processing, and the results are shown in Tables 1to 3.

From the above research results, it can be seen that the deep learning-based digital creative sports industry development research model proposed in this article has good results and has a certain role in promoting the development of the digital creative sports industry.

## 5. Conclusion

The digital entrepreneurial sports cultural creative industry is a part of the cultural industry. It has the general characteristics of cultural industry development, and as a member of the cultural industry, it has its own characteristics and development methods. The digital entrepreneurial sports cultural creative industry is the same as other creative industries in that it is combined with other industrial forms through certain technologies to form a new development form with its own characteristics. The digital entrepreneurial sports cultural creative industry presents the characteristics of high knowledge and intelligence. This industry takes culture and creative ideas as the core and is a specific product of human knowledge, wisdom, and inspiration. Secondly, the digital entrepreneurial sports cultural creative industry has the characteristics of high added value. The digital entrepreneurial sports cultural and creative industry as the “sum of industrialized activities” is an industrialized activity in which sports advertising, sports animation, sports publishing, sports entertainment, sports event TV copyright, sports event ticket sales, and sports tourism are the contents. This article uses deep learning technology to study the development of the digital creative sports industry, builds an intelligent model, and combines simulation data for model research to provide a theoretical reference for the subsequent development of the digital creative sports industry.

## Figures and Tables

**Table 1:** Evaluation of the market analysis effect of the digital creative sports industry development research model.

Number	Market analysis	Number	Market analysis	Number	Market analysis

1	84.70	16	90.62	31	88.10
2	85.59	17	93.91	32	86.97
3	89.98	18	92.72	33	90.68
4	91.73	19	87.83	34	86.54
5	85.35	20	83.29	35	92.19
6	93.42	21	84.85	36	89.20
7	91.74	22	86.62	37	87.64
8	92.80	23	90.87	38	92.35
9	92.26	24	83.10	39	83.17
10	93.55	25	87.66	40	92.32
11	92.54	26	91.33	41	85.41
12	84.77	27	85.99	42	83.48
13	93.18	28	88.82	43	86.60
14	88.09	29	89.62	44	86.92
15	88.81	30	86.13	45	84.35

**Table 2:** Evaluation of the effect of data processing on the development research model of digital creative sports industry.

Number	Data processing	Number	Data processing	Number	Data processing

1	85.79	16	80.71	31	77.26
2	83.72	17	84.67	32	88.33
3	80.56	18	76.82	33	85.95
4	85.20	19	78.21	34	80.15
5	85.07	20	77.61	35	82.50
6	76.33	21	88.47	36	84.17
7	78.49	22	83.85	37	79.78
8	76.82	23	85.21	38	75.43
9	85.67	24	78.40	39	87.34
10	86.60	25	77.64	40	87.86
11	76.64	26	82.52	41	75.77
12	79.22	27	82.41	42	82.61
13	81.76	28	89.33	43	87.54
14	89.60	29	88.53	44	85.39
15	80.66	30	87.18	45	76.02

**Table 3:** Evaluation of the decision-making effect of the development research model of the digital creative sports industry.

Number	Development decision	Number	Development decision	Number	Development decision

1	89.66	16	76.95	31	81.08
2	84.40	17	90.87	32	75.77
3	77.28	18	86.21	33	77.60
4	84.20	19	82.43	34	81.59
5	79.52	20	79.96	35	75.50
6	75.29	21	88.18	36	75.65
7	90.59	22	78.71	37	81.41
8	82.43	23	81.18	38	83.74
9	91.70	24	74.52	39	79.39
10	84.06	25	87.90	40	82.61
11	75.10	26	78.46	41	86.00
12	89.11	27	80.65	42	86.42
13	78.11	28	84.47	43	78.49
14	87.20	29	87.10	44	89.64
15	87.40	30	89.44	45	90.90

## Data Availability

The labeled datasets used to support the findings of this study are available from the corresponding author upon request.

## References

[1] Marwan I. I. S., Rohayati N. I. A. (2020). Model of physical education in digitalization era: improving thinking activities and physical conditions. *Systematic Reviews in Pharmacy*.

[2] Stojanovski T., Partanen J., Samuels I., Sanders P., Peters C. (2020). Viewpoint: city information modelling (CIM) and digitizing urban design practices. *Built Environment*.

[3] Matsui K., Azuma A. (2021). The effect of self-analysis of the movement of running long jump using a strobe picture on college male students’ performance in PE class. *Advances in Physical Education*.

[4] Inec Z. F., Akpinar E. (2020). Digitizing and interpreting the world map drawn by Kashgarli Mahmud: constructing information using evidence based political literacy. *Review of International Geographical Education Online*.

[5] Yi A. K. J., Yean T. S., Ann T. B. (2021). Digitizing Malaysia: examining the role of ICT in Malaysia’s exports of goods and services. *Thailand and The World Economy*.

[6] Ellwood E. R., Kimberly P., Guralnick R. (2018). Worldwide engagement for digitizing biocollections (WeDigBio): the biocollections community’s citizen-science space on the calendar. *BioScience*.

[7] Mulligan D., Lohse K. R., Hodges N. J. (2016). An action-incongruent secondary task modulates prediction accuracy in experienced performers: evidence for motor simulation. *Psychological Research*.

[8] Khudolii O. M., Ivashchenko O. V., Iermakov S. S., Rumba O. G. (2016). Computer simulation of junior gymnasts’ training process. *Science of Gymnastics Journal*.

[9] Tivener K. A., Gloe D. S. (2015). The effect of high-fidelity cardiopulmonary resuscitation (CPR) simulation on athletic training student knowledge, confidence, emotions, and experiences. *Athletic Training Education Journal*.

[10] Ivashchenko O. V., Kapkan O. O. (2015). Simulation of process of 14-15 years old girls’ training of light athletic and gymnastic exercises. *Pedagogics, psychology, medical-biological problems of physical training and sports*.

[11] Owen P. D., King N. (2015). Competitive balance measures in sports leagues: the effects of variation in season length. *Economic Inquiry*.

[12] Yang J. (2019). The simulation of table tennis during the course of sports. *Caribbean Journal of Science*.

[13] Bulat M., Korkmaz Can N., Arslan Y. Z., Herzog W. (2019). Musculoskeletal simulation tools for understanding mechanisms of lower-limb sports injuries. *Current Sports Medicine Reports*.

[14] Payne T., Mitchell S., Bibb R., Waters M. (2015). The evaluation of new multi-material human soft tissue simulants for sports impact surrogates. *Journal of the Mechanical Behavior of Biomedical Materials*.

[15] Lopatiev A., Ivashchenko O., Khudolii O., Pjanylo Y. (2017). Systemic approach and mathematical modeling in physical education and sports. *Journal of Physical Education and Sport (JPES)*.

[16] Bennour N. (2015). Teaching practices and student action in physical education classes: perspectives for teacher education. *Creative Education*.

[17] Sghaier D., Elandoulsi S., Mami M., Bouassida A. (2015). Physical education teacher’s training in swimming under the joint didactic action. *Creative Education*.

[18] Souza Í. M. M. d., Silva C. L. d. (2015). Body practices and Brazilian culture: pedagogical contributions to physical education professionals. *Procedia - Social and Behavioral Sciences*.

[19] Cutforth N., Belansky E. S. (2015). A community-engaged approach to translating research into practice: a physical education story. *Progress in Community Health Partnerships: Research, Education, and Action*.

[20] Hwang E.-R., Kim T.-Y. (2018). Intensification of the education of public health, hygiene, and martial arts during the Japanese colonial period (1937-1945). *Journal of Exercise Rehabilitation*.

[21] Murase D., Yokoyama K., Fujii K., Hasegawa Y. (2016). Baseball catching patterns differ according to task constraints. *Advances in Physical Education*.

